# Analysis of possible factors of vocal interference during the teaching activity

**DOI:** 10.11606/S1518-8787.2017051000092

**Published:** 2017-12-04

**Authors:** Bárbara Gabriela Silva, Tiago Visacre Chammas, Marcia Simões Zenari, Renata Rodrigues Moreira, Alessandra Giannella Samelli, Kátia Nemr

**Affiliations:** I Universidade de São Paulo. Faculdade de Medicina. Departamento de Fisioterapia, Fonoaudiologia e Terapia Ocupacional. São Paulo, SP, Brasil; II Universidade de São Paulo. Hospital Universitário. São Paulo, SP, Brasil

**Keywords:** School Teachers, Voice Disorders, epidemiology, Noise, adverse effects, Hearing Loss, Risk Factors, Working Conditions, Professores Escolares, Distúrbios da Voz, epidemiología, Ruído, efeitos adversos, Perda Auditiva, Fatores de Risco, Condições de Trabalho

## Abstract

**OBJECTIVE:**

To measure the risk of dysphonia in teachers, as well as investigate whether the perceptual-auditory and acoustic aspects of the voice of teachers in situations of silence and noise, the signal-to-noise ratio, and the noise levels in the classroom are associated with the presence of dysphonia.

**METHODS:**

This is an observational cross-sectional research with 23 primary and secondary school teachers from a private school in the municipality of São Paulo, Brazil, divided into the groups without dysphonia and with dysphonia. We performed the following procedures: general Dysphonia Risk Screening Protocol (General-DRSP) and complementary to speaking voice - teacher (Specific-DRSP), voice recording during class and in an individual situation in a silent room, and measurement of the signal-to-noise ratio and noise levels of classrooms.

**RESULTS:**

We have found differences between groups regarding physical activity (General-DRSP) and particularities of the profession (Specific-DRSP), as well as in all aspects of the perceptual-auditory vocal analysis. We have found signs of voice wear in the group without dysphonia. Regarding the vocal resources in the situations of noise and silence, we have identified a difference for the production of abrupt vocal attack and the tendency of a more precise speech in the situation of noise. Both the signal-to-noise ratio and the room noise levels during class were high in both groups.

**CONCLUSIONS:**

Teachers in both groups are at high risk for developing dysphonia and have negative vocal signals to a greater or lesser extent. Signal-to-noise ratio was inadequate in most classrooms, considering the standards for both children with normal hearing and with hearing loss, as well as equivalent noise levels.

## RESUMO

**OBJETIVO:**

Mensurar o risco de disfonia em professores, bem como investigar se os aspectos vocais perceptivo-auditivos e acústicos em situação de ruído, a relação sinal-ruído e os níveis de ruído em sala de aula estão associados à presença de disfonia.

**MÉTODOS:**

Pesquisa transversal observacional com 23 professores da educação infantil e ensino fundamental de uma escola particular do município de São Paulo divididos nos grupos sem disfonia e com disfonia. Foram realizados os seguintes procedimentos: protocolo de rastreio de risco de disfonia geral (PRRD-Geral) e complementar para voz falada - professor (PRRD-Específico), gravação da voz durante aula e em situação individual em sala silenciosa, medição da relação sinal-ruído e dos níveis de ruído das salas de aula.

**RESULTADOS:**

Foram encontradas diferenças entre os grupos quanto à atividade física (PRRD-Geral) e particularidades da profissão (PRRD-Específico), bem como em todos os aspectos da análise vocal perceptivo-auditiva. No grupo sem disfonia, foram encontrados sinais de desgaste da voz. Quanto aos recursos vocais nas situações de ruído e silêncio, identificamos diferença para produção de ataque vocal brusco e tendência de uma fala mais precisa na situação-ruído. Tanto a relação sinal-ruído quanto os níveis de ruído das salas durante a aula foram elevados nos dois grupos.

**CONCLUSÕES:**

Os professores dos dois grupos estão expostos a riscos elevados para o desenvolvimento de disfonias e apresentam sinais vocais negativos em maior ou menor grau. A relação sinal-ruído apresentou-se inadequada em grande parte das salas de aula, considerando-se as normas tanto para crianças com audição normal quanto para aquelas com perda auditiva, assim como os níveis de ruído equivalentes.

## INTRODUCTION

Studies on the voices of teachers show a high rate of dysphonia in this professional class^3^ ^,^ ^10^ ^,^ ^24^. The severity of dysphonia can negatively interfere in the viability of the teaching work, with consequent hindering of the comprehension of students^22^ and, in more serious cases, the medical leave of these professionals from their duties^19^.

Among the working conditions that permeate the health-disease process of teachers, we highlight the long working hours, prolonged and excessive vocal use^5^, and the high and constant levels of noise in classrooms^11^, which are above that recommended by regulation NBR 10152, between 40 dB and 50 dB(A)^a^.

During classes, excessive noise, in addition to interfering with the attention and concentration of teachers^11^ and hindering the auditory processing of essential information for the development of activities, competes with the vocal production, which may lead to inappropriate voice adjustments, increasing the risk of dysphonia^20^ ^,^ ^24^.

An acoustic interference factor in the classroom, and an important tool to monitor both the noise and the voices of teachers, is the signal-to-noise ratio (SNR)^4^, calculated from the difference, in decibels (dB), between the level of the signal of interest (in this case, the voice of the teacher) and the background noise intensity of the environment; it can vary according to the distance and intensity of the signal of interest, the reverberation of the environment, and the background noise^8^.

The investigation of both the risk of dysphonia and the noise during the teaching activity aims to assist in the targeting of programs and activities for the promotion and prevention of vocal disorders, as well as the necessary modifications in the organization and conditions of the work to reduce the number of medical leaves and also to improve the teaching and the quality of life of those involved.

The objective of this study is to measure the risk of dysphonia in teachers, as well as investigate whether the perceptual-auditory and acoustic aspects of the voice of teachers in the situation of silence and noise, the signal-to-noise ratio, and the noise levels in the classroom are associated with the presence of dysphonia.

## METHODS

This is an observational cross-sectional research approved by the Research Ethics Committee of the Faculdade de Medicina of the Universidade de São Paulo (Process 169/15), carried out in a private primary and secondary school in the municipality of São Paulo.

Twenty-three female teachers, with a mean age of 37.26 years (SD = 7.0), signed the informed consent.

We carried out the following procedures at the school itself:

- Application of the General Dysphonia Risk Screening Protocol (General-DRSP)^15^ and the Complementary Dysphonia Risk Screening Protocol for Speaking Voice Professional, Teacher (Specific-DRSP)^b^, for the identification of vocal complaints, general and occupational life habits, among others. The protocols were explained by the researchers and filled out by the teachers in their presence.
- Recording of the voice in a silent environment (situation of silence) for later perceptive- auditory and acoustic vocal analysis: sustained vowels, phrases, and spontaneous speech according to the CAPE-V protocol^c^. We used the equipment ZOOM H4 placed perpendicularly at 30 cm from the mouth of the teacher.
- Voice recording with the ZOOM H4 recorder positioned in the middle of the classroom (situation of noise) for 15 minutes of effective speech (situation of expository class).
- Together with the recording of the teachers in the classrooms, we used a dosimeter, SVANTEK (model SV102, Poland), in the middle of the classroom, to measure the sound pressure levels (maximum, minimum, and equivalent noise levels [Leq]), for 15 minutes.

### Data Analysis

We obtained a final score from the screening protocols, which consists in adding the values of the partial scores of each subitem; the General-DRSP ranges from zero to 131 and the Specific-DRSP ranges from zero to 56 for men and from zero to 60 for women^15^. The higher the value obtained, the greater the risk of dysphonia^15^ ^,^ ^b^.

The perceptual-auditory analysis of the voice recordings obtained in silence was carried out by a speech-language voice expert with extensive experience in evaluations with CAPE-V and high internal reliability of judgment, as measured in an earlier study^15^. From the value of the general degree of vocal change, teachers were divided into two groups: without dysphonia (GWOD), for values between zero and 34, or with dysphonia (GWD), for values greater than 34^12^. We carried out the acoustic evaluation of the same sample using the free program Praat, considering the automatic measurements of fundamental frequency (normality: 150 Hz to 250 Hz), jitter (0.30% to 0.50%), shimmer (3% and 5%), and harmonics-to-noise ratio (22 dB)^2^ ^,^ ^17^ ^,^ ^d^.

The voices captured in the situation of silence and situation of noise were analyzed by two of the researchers by consensus, being classified from the vocal resources^9^: type of voice (neutral, altered), vocal attack (isochronic, abrupt, aspirated), loudness (very loud, average, soft), pitch (low, average, high), resonance (balanced, oral, laryngopharyngeal, nasal, hyponasal), articulation (precise, reduced), speed of the speech (slow, average, accelerated), and pneumophonoarticulatory coordination (presence, absence).

With the voice recorded in class, we measured the SNR of each classroom using the program Audacity 2.0.6, the Canadian protocol E-Ramp-Inc. In order to classify the SNR as adequate or inadequate, we considered the cutoff values of +15 dB for classrooms of children with normal hearing (ASHA, 2000)^e^ and +20 dB for children with hearing loss (British Association of Teachers of the Deaf, 2009)^f^.

In relation to the classification as adequate or increased of the sound pressure levels in the classroom, we used the NBR 10152 (ABNT, 2000)^a^, which considers ambient noise of 40 to 50 dB(A) as acceptable for classrooms.

### Statistical Analysis

We used descriptive measures and the unpaired ANOVA and chi-square tests (exact test mid P) with a significance level of 0.05.

## RESULTS

As for the division of the groups from the values of the General Degree of the CAPE-V, we obtained the GWOD with eleven participants (mean score of 20.81, SD = 10.1) and the GWD with twelve individuals (mean score of 52.91, SD = 12.43).

Regarding the General-DRSP, we found a difference only for the partial score of the subitem of regular physical activity (Table 1), with a lower occurrence in the GWD. For Specific-DRSP, we found a difference in the subitem of specificities of the profession (Table 1), in which GWOD had a higher score. There was no difference between the groups for the final score of General-DRSP and Specific-DRSP.

**Table 1:** Mean, standard deviation, minimum, and maximum for the scores of the General-DRSP and Specific-DRSP for the groups with and without dysphonia.

Characteristic	Group	Average	SD	Minimum	Maximum	p^a^
General-DRSP						
Visual analogue scale	GWOD	3.38	2.37	0	6.3	0.066
	GWD	5.24	2.24	1	8.3	
Previous vocal disorders	GWOD	1.64	0.92	0	2	0.505
	GWD	1.83	0.39	1	2	
Current symptoms	GWOD	20	13.17	2	39	1
	GWD	20.16	7.75	9	33	
Vocal use outside work	GWOD	2.27	1.27	0	4	0.534
	GWD	2.42	0.79	1	4	
Diet	GWOD	2.36	1.36	0	4	0.578
	GWD	2.08	0.99	1	4	
Hydration	GWOD	1.73	1.27	0	3	0.584
	GWD	1.42	1.38	0	3	
Medication	GWOD	0.09	0.3	0	1	0.336
	GWD	0.25	0.45	0	1	
Smoking	GWOD	0.18	0.4	0	1	0.609
	GWD	0.33	0.89	0	3	
Sleep	GWOD	1.36	1.36	0	3	0.755
	GWD	1.5	0.52	1	2	
Diseases	GWOD	0.91	0.94	0	2	0.348
	GWD	1.33	1.15	0	3	
History of vocal disorder in the family	GWOD	0.18	0.4	0	1	0.134
	GWD	0	0	0	0	
Family dynamics	GWOD	0	0	0	0	0.351
	GWD	0.08	0.29	0	0	
Physical activity	GWOD	0.36	0.5	0	1	0.004^b^
	GWD	0.92	0.29	0	1	
Leisure	GWOD	0	0	0	0	0.171
	GWD	0.17	0.39	0	1	
Final score	GWOD	36.47	16.26	11	62	0.572
	GWD	39.74	10.75	25	58	
Specific-D						
Usage time	GWOD	2.73	1.01	1	4	0.596
	GWD	2.92	0.67	2	4	
Profession	GWOD	11.72	0.47	11	12	0.043^b^
	GWD	10.91	1.16	9	12	
Environmental conditions	GWOD	8.64	1.03	8	11	0.534
	GWD	9.25	3.08	2	14	
Medical leave	GWOD	0.27	0.47	0	1	0.249
	GWD	0.08	0.29	0	1	
Smoking	GWOD	0	0	0	0	0.171
	GWD	0.5	1.17	0	3	
Alcohol consumption	GWOD	0.27	0.47	0	1	0.921
	GWD	0.25	0.45	0	1	
Consumption of drugs	GWOD	0	0	0	0	1
	GWD	0	0	0	0	
Dental prosthesis	GWOD	0.09	0.3	0	1	0.306
	GWD	0	0	0	0	
Hormonal factors	GWOD	0.64	0.5	0	1	0.304
	GWD	0.83	0.39	0	1	
Final score	GWOD	24.36	2.01	22	27	0.743
	GWD	34.75	3.44	19	30	

GWOD: group without dysphonia; GWD: group with dysphonia

a ANOVA test.

b p < 0.05

We found no differences between the groups in any of the acoustic vocal aspects evaluated. Regarding CAPE-V, there was a difference between groups in all aspects, with a greater change for the GWD (Table 2).

**Table 2:** Mean, standard deviation, minimum, and maximum for acoustic and perceptual-auditory analysis for groups with and without dysphonia.

Characteristic	Group	Average	SD	Minimum	Maximum	p^a^
Analysis of acoustic measurements						
Fundamental frequency (Hz)	GWOD	191.19	22.37	164.45	231.92	0.702
	GWD	195.19	26.85	142.41	244.24	
Jitter (%)	GWOD	0.39	0.21	0.21	0.95	0.166
	GWD	0.28	0.16	0.13	0.73	
Shimmer (%)	GWOD	4.41	1.99	1.78	7.38	0.794
	GWD	4.19	2.09	1.26	9.44	
Harmonics-to-noise ratio (dB)	GWOD	18.97	5.65	10.19	27.42	0.889
	GWD	18.65	4.1	10.23	24.84	
Auditory-perceptual analysis - CAPE-V						
Roughness	GWOD	13.64	10.89	0	34	0.0005^b^
	GWD	43.42	21.42	4	71	
Breathiness	GWOD	19.91	9.53	5	33	0.0025^b^
	GWD	45.25	22.74	0	73	
Strain	GWOD	1.73	3.47	0	11	0.0001^b^
	GWD	28.5	18.83	0	61	
Pitch	GWOD	8.73	10.08	0	26	0.0093^b^
	GWD	27.17	19.01	0	73	
Loudness	GWOD	1.27	4.22	0	14	0.0229^b^
	GWD	12.67	14.84	0	37	

GWOD: group without dysphonia; GWD: group with dysphonia

a ANOVA test.

b p < 0.05

As for the averages of the SNR obtained during classes, we verified a tendency to a difference between groups (p = 0.096), with a higher average for the GWD (Table 3). When evaluating each classroom separately, we verified that the SNR was altered in nine of them (39%), considering the standard for normal hearing, and in 23 classrooms (95%), considering the standard for hearing loss.

**Table 3:** Mean, standard deviation, minimum, and maximum for the signal-to-noise ratio for groups with and without dysphonia.

Characteristic	Group	Average	SD	Minimum	Maximum	p*
Signal-to-noise ratio (dB)	GWOD	15.1	2.83	11.1	19.9	0.096
	GWD	17.1	2.68	13.1	21.6	

GWOD: group without dysphonia; GWD: group with dysphonia

* ANOVA test.

When we evaluated the vocal resources in the two different situations (noise and silence), we observed that a greater number of teachers performed an abrupt vocal attack in the situation of noise when compared to the situation of silence. In addition, teachers showed a tendency to make a more accurate articulation of speech sounds in the situation of noise (p = 0.052) (Table 4).

**Table 4:** Number of teachers (n) in each situation (silence and noise) and in each classification of vocal resources.

Variable	Classification	Silence (n)	Noise (n)	p^a^
Type of voice	Neutral	18	19	0.729
	Non-neutral, diverted, or altered	5	4	
Vocal attack	Isocronic	23	18	0.024^b^
	Abrupt	0	5	
Loudness	Average	16	11	0.15
	Soft or very loud	7	12	
Pitch	Average	14	16	0.155
	Low or high	9	7	
Resonance	Balanced	10	13	0.397
	Unbalanced	13	10	
Articulation	Precision	17	22	0.052
	Reduced	6	1	
Speed of the speech	Adequate	23	22	0.5
	Increased	0	1	
Speech-breathing coordination	Presence	20	23	0.116
	Absence	3	0	

a Chi-square test.

b p < 0.05

Based on the relationship between noise exposure and abrupt vocal attack, we carried out a risk analysis for this factor. We identified that the risk of a vocal attack in the situation of noise corresponds to 21.7% (95%CI 9.2-42.3). In the silent environment, the risk was non-existent (95%CI 0-16.9).

As for the average noise levels measured in the classrooms, we identified no difference between the groups for any measure, even though the values in the classrooms of the GWD were higher. The averages of Leq for both groups demonstrate that classroom noise is not suitable for most of them. Only two classrooms (8.7%) in the GWOD had noise below 50 dB(A) during class.[Table t5]

**Table 5 t5:** Mean and standard deviation for noise levels (Leq, minimum, and maximum in dBA) for groups with and without dysphonia.

Variable	Group	Average	SD	p*
Leq	GWOD	68.91	16.63	0.180
	GWD	76.22	7.32	
Maximum	GWOD	80.25	23.52	0.242
	GWD	88.97	8.52	
Minimum	GWOD	53.16	7.05	0.361
	GWD	55.18	2.48	

GWOD: group without dysphonia; GWD: group with dysphonia

* ANOVA test.

## DISCUSSION

The voice of teachers is a much-researched subject, but few studies relate environmental issues to the risk of dysphonia^7^ ^,^ ^13^. The evaluation of this risk is an important tool for actions that promote health in the school environment, which can guide a process of awareness of those involved, as well as change habits and the environment to improve the quality of life. In addition, actions to promote health in this environment favor the earlier diagnosis of dysphonia^1^.

The group of participants in this research was composed of female teachers with a mean age of 37.26 years. It is known that there is a greater frequency of women in this profession; however, the age group is quite varied^3^ ^,^ ^5^ ^,^ ^7^ ^,^ ^10^ ^,^ ^19^ ^,^ ^22^ ^,^ ^24^ ^,^ ^b^. When compared to men, women are more prone to vocal disorders because of the laryngeal configuration^13^ ^,^ ^18^.

Using the General-DRSP and the Specific-DRSP, we observed in this study that, regardless of dysphonia, teachers are exposed to similar risks for dysphonia, as found in a previous study^b^.

Regarding General-DRSP, there was a difference between the groups only for the regular practice of physical activity, greater in the group without dysphonia. Vocal strain was lower in the group without dysphonia. Those who practice physical activity may have lower levels of stress and body strain^14^ and, consequently, vocal strain, which suggests a relationship between physical inactivity and dysphonia^1^. In addition, the practice of physical exercises reduces the symptoms of depression and anxiety^14^, which would positively impact the vocal production without strain and the quality of life of teachers. We emphasize that the practice of physical activity depends on the routine and life habits established by the individual and can be reinforced in actions of health promotion.

Regarding Specific-DRSP, the groups differed in the particularities of the current profession, and the GWOD had the highest score. Looking more closely at these data, we noted that only teachers considered as regents were present in the GWOD and expert teachers (arts, music, and computer science) were present only in the GWD. When calculating the score, the regent teacher receives one more point than the expert because it is often considered that the expert allocates part of the class time to the practice of the students and, therefore, they supposedly have less expository lesson time than the regent teacher, which would be positive for vocal well-being. However, if all the expert teachers were in the GWD, this factor may be being minimized by other factors of higher risk for dysphonia. We should also consider that children can get more agitated in these classes, which could lead the expert teacher to retake the limits using the voice. Studies with other schools will allow for broader analyses and may even indicate the need to revise the calculation of the score for Specific-DRSP.

Another aspect of this subitem that had a higher score in the GWOD was the number of years working as a teacher at the current level of education. Contrary to what we can find in the literature^15^ ^,^ ^25^, teachers with no vocal disorder indicated more years teaching in the classroom when compared to teachers of the GWD. This data was also observed in another study^7^, in the characterization of the sample, in which only 33% of the teachers with vocal disorders had more than ten years of work. This finding may indicate a positive adaptation of these professionals over time or even some training received, which can also be better explored in future studies. Regarding the level of education, most teachers of the GWOD work in secondary education and half of the teachers of the GWD work in primary education. A study carried out with primary teachers^23^ has found a higher prevalence of dysphonia compared to other studies with teachers of secondary education, high school, and college, which may indicate that the particularities of the work along this age group should be one more factor to be considered.

Thus, taking into account the particularities of the work, the changes that can occur each semester in the schedule of teachers regarding the hours/class and that would justify a periodic monitoring to evaluate possible tendencies of increase or decrease of risks must be considered; in addition, this control could contribute with the analyses about possible vocal fatigue at the end of each semester.

Although we observed no differences between the groups regarding the final score of the General-DRSP and Specific-DRSP, we observed a higher mean in the score of the GWD, which could be better evidenced in studies with larger samples.

The averages obtained in the score for General-DRSP for both groups were above the cutoff point for women, which is 29.25^15^. This represents a high risk of dysphonia for teachers with and without a vocal disorder, corroborating other studies^1^ ^,^ ^b^.

The reduction of risks requires changes in the conditions and organization of the work and depends on mobilizations from higher instances^5^. From this study, we observed that, regardless of the presence and degree of vocal change, teachers are daily exposed to high occupational risks for dysphonia^1^ ^,^ ^7^ ^,^ ^b^.

Concerning the acoustic analysis of voices recorded in a quiet environment, according to the parameters evaluated, no differences were found between the groups. In a pre-study with 28 educators, with acoustic vocal analysis performed using the same software, groups of teachers with adequate voices, voices altered to a mild degree, and voices altered to a moderate degree were compared^24^. As in this research, the authors^24^ have not observed differences for acoustic parameters of fundamental frequency, shimmer, and harmonics-to-noise ratio. However, they have found differences in jitter. In this study, although there was no difference between the groups in relation to the vocal parameters in the acoustic analysis, the average found in the GWD was below the reference values. A research with 99 teachers, with acoustic analysis of voices made using another software (Multi-Dimensional Voice Program Advanced), has found measures of jitter, shimmer, and fundamental frequency above normality^21^. The values of harmonics-to-noise ratio were lower than expected in the two groups, which indicate that voices considered as unchanged in the GWOD are presenting more noise than expected, which may be a sign of vocal fatigue^24^. This data shows the importance of combining perceptual-auditory and acoustic analysis for greater detail.

In the perceptual-auditory vocal analysis using the CAPE-V, we found differences in all aspects evaluated when comparing the two groups, with changes in the GWD. Nevertheless, the GWOD presented breathiness and roughness, although reduced and not configuring dysphonia, which may be associated with the changes observed in the acoustic measures such as jitter and harmonics-to-noise ratio discussed above.

The greater presence of roughness in teachers with dysphonia may be related to mass changes in the vocal folds, such as vocal nodules that are commonly found in this professional category^22^ and more frequently in women, as well as breathiness, because of the laryngeal configuration in itself, prone to existence of glottic chink^18^. These data emphasize the importance of evaluating the laryngeal conditions of teachers with dysphonia.

The vocal changes found reinforce the findings of other studies^21^ ^,^ ^22^ and justify the actions of health promotion and vocal well-being that improve the voice quality of these professionals. Simple interventions, such as vocal warm-up practice and respiratory training, have positive results^16^.

Regarding the SNR, there was a higher average tendency in the GWD, suggesting that the voice is more intense in the classrooms of teachers with dysphonia than in the other classrooms. This data is similar to other studies^20^ ^,^ ^24^ and represents an important occupational risk factor for these professionals.

In the situation of noise, we observed the use of abrupt vocal attack, with a risk of 21.7% in its use in classes. The abrupt vocal attack consists of an inadequate vocal pattern related to phonotrauma, in which there is a rapid and complete adduction of the vocal folds at the beginning of phonation. This condition may be followed by muscle strain and it signals increased effort when frequent^2^. In order to be understood in classrooms, teachers can change their habitual vocal pattern, not always with adequate adjustment for their laryngeal conditions, as is the case of abrupt vocal attack^20^ ^,^ ^24^.

A protective factor observed in both groups was the tendency to articulate speech sounds more accurately in the situation of noise. In addition, both groups maintained adequate pitch, speed of the speech, and speech-breathing coordination when exposed to noise. Both results suggest a natural positive adjustment or prior training.

Among the possible occupational risks, this study emphasized the investigation of the noise levels present in classrooms, as well as the analysis of the signal-to-noise ratio. The monitoring of these two measures in the school context is fundamental to guide actions that minimize damage to the voice of teachers. In addition, these levels, when above recommended, may interfere not only with teacher performance but also with student learning^11^.

Regarding the noise present in classrooms, there was no difference between the groups for the averages of equivalent noise, although the values of the classrooms of the GWD were higher. Moreover, only two classrooms (8.7%) presented noise below what is proposed during classes in the GWOD^7^ ^,^ ^a^. The signal-to-noise ratio was lower than that recommended for children with normal hearing^e^ in approximately 39% of the classrooms, and for children with hearing loss^f^ in approximately 95% of them. The high levels of noise found reinforce the current situation of schools in Brazil^7^ ^,^ ^11^ ^,^ ^13^.

With the reduction of noise levels in classrooms, the signal-to-noise ratio increases without the teacher having to make adjustments in the vocal pattern, which facilitates the understanding of students and decreases the risk of dysphonia.

It is important to point out the need for new research to investigate and emphasize the importance of the signal-to-noise ratio also in the monitoring of vocal problems, since the signal-to-noise ratio can be an important ally to control the noise intensity and listening situation offered in classrooms.

Programs in this direction could enable preventive measures, as well as the development of strategies that would empower teachers and school staff as health promoters.

## Footnotes

a Associação Brasileira de Normas Técnicas. NBR 10152: níveis de ruído para conforto acústico. Rio de Janeiro: ABNT; 2000

b Sampaio AP, Simões-Zenari M, Nemr NK. Aplicabilidade de protocolo de rastreio de risco de disfonia em professores. São Paulo: Faculdade de Medicina da Universidade de São Paulo; 2015. Trabalho de Conclusão de Curso de Fonoaudiologia.

c American Speech-Language Hearing Association, ASHA Division 3 - Voice and Voice Disorders; University of Pittisburg, Department of Communication Science and Disorders. A Consensus Auditory - Perceptual Evaluation of Voice (CAPE -V). Pittisburgh: ASHA; 2002 [cited 2017 Oct 23]. Available from: https://csd.wisc.edu/slpgames/capev_activities/capev_Instructions.pdf

d Leão SHS. Análise espectrográfica acústica de vozes rugosas, soprosas e tensas [dissertação]. São Paulo: Universidade Federal de São Paulo - UNIFESP; 2008

e Crandell CC, Smaldino JJ. Classroom acoustics for children with normal hearing and with hearing impairment. *Lang Speech Hear Serv Sch.* 2000;31(4):362- 70. https://doi.org/10.1044/0161-1461.3104.362

f Glasgow M. Classroom acoustics: recommended standards: classroom acoustic standards for children with sensori-neural hearing loss. Cheshire (UK): The British Association of Teachers of the Deaf; 2009.
